# Sex specific familial risk in lung cancer through changing histologies in Sweden

**DOI:** 10.1002/ijc.35431

**Published:** 2025-03-29

**Authors:** Kari Hemminki, Frantisek Zitricky, Kristina Sundquist, Jan Sundquist, Asta Försti, Akseli Hemminki

**Affiliations:** ^1^ Biomedical Center, Faculty of Medicine Charles University Pilsen Pilsen Czech Republic; ^2^ Division of Cancer Epidemiology German Cancer Research Center (DKFZ) Heidelberg Germany; ^3^ Center for Primary Health Care Research Lund University Malmö Sweden; ^4^ University Clinic Primary Care Skåne Skåne Sweden; ^5^ Department of Family and Community Medicine, McGovern Medical School The University of Texas Health Science Center Houston Texas USA; ^6^ Hopp Children's Cancer Center (KiTZ) Heidelberg Germany; ^7^ Division of Pediatric Neurooncology German Cancer Research Center (DKFZ), German Cancer Consortium (DKTK) Heidelberg Germany; ^8^ Cancer Gene Therapy Group, Translational Immunology Research Program University of Helsinki Helsinki Finland; ^9^ Comprehensive Cancer Center Helsinki University Hospital Helsinki Finland

**Keywords:** adenocarcinoma, age of onset, incidence trend, proband, sibling risk

## Abstract

Familial clustering of lung cancer (LC) is related to shared smoking habits but the contribution of other potential factors such as sex or histology is not well known, and these are the subjects of the present study in Sweden. Family relationships (from Multigeneration register) and diagnosed cancers (from Cancer registry) were obtained from the national registers from 1961 to 2021. The overall familial risk for LC was constant from the 1990s but the male familial risk decreased while the female familial risk doubled at the same time when female incidence doubled. The female familial risk for mother‐daughter pairs was higher (SIR = 2.2 [2.0–2.3], *N* = 716) than for father‐son pairs (SIR = 1.6 [1.5–1.8], *N* = 962). The histology‐specific familial risks for adenocarcinoma, squamous cell carcinoma, small cell and large cell carcinoma were highest for concordant histology but also present for discordant histology. The number of family members diagnosed with LC was a strong determinant of familial risk. The novel results showed that familial risk of LC depends on the background incidence of LC and is higher for women compared to men. We demonstrated further an increased familial risk for each of the four histological types of LC which was higher for concordant than discordant histologies but was even detected between discordant histologies suggesting that LC histology is not a genetic trait.

## Graphical abstract

What's new?

Lung cancer is a complex disease arising from genetic and environmental factors. In this study, using data from family and cancer registries in Sweden, the authors investigated familial risks in lung cancer, with focus on sex‐ and time‐related changes and correlations with histology. Analyses reveal distinct sex‐specific shifts in lung cancer risk since the 1990s, with declines in male and increases in female familial risks. Familial risk was identified in the four main histological lung cancer types, indicating that histology is modified by sex‐ and time‐dependent factors. Familial risk was further influenced by the number of family members affected.

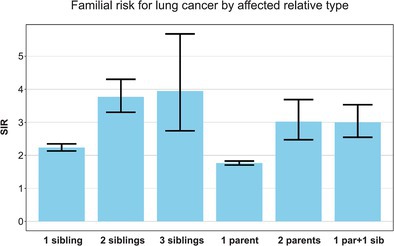

**Abbreviations**
CI: confidence interval
ICD: incternational classification of deseases
LC: lung cancer
PAD: pathologic‐anatomic diagnosis
SCC: squamous cell carcinoma
SIR: standardized incidence ratio
US: United States
WHO: World Health Organization

## 1 INTRODUCTION

Global literature on lung cancer (LC) incidence rates shows that these follow prevalence rates of cigarette smokers with a lag time of 20–30 years.^1^, ^2^, ^3^ In industrial countries the main LC histology for men has been squamous cell carcinoma (SCC) which was taken over later by adenocarcinoma, the type that has been the main female histology.^4^ In US men, the total LC incidence culminated before 1990 and in women at around 2005.^5^ White male SCC reached a maximal incidence at around 1980 and in women after 1990; male adenocarcinoma incidence plateaued after 1990 and female after 2010.^5^ For rarer histological types, small cell and large cell LC the rates for both sexes culminated at around 1990.^5^ In USA, the incidence rate ratio (male/female) for all LC in white population was close to 3.0 in the 1970s but by 2010 it declined to 1.3.^5^ In Sweden, male LC incidence peaked in 1982 and that for women in 2018, shortly before the epochal crossing of the male and female LC incidence rates.^6^ The incidence for male SCC plateaued at around 1980 having exceeded adenocarcinoma incidence over 3‐fold.^7^ The plateauing of the female SCC incidence was much later, in 2010 (https://sdb.socialstyrelsen.se/if_can/val.aspx). The incidence in adenocarcinoma increased steadily and the female incidence exceeded the male incidence after year 2000 reaching a maximum in 2016, 5 years after the male maximum (https://sdb.socialstyrelsen.se/if_can/val.aspx). Small cell LC was first reported in Sweden in 1985 when its share of all LC was close to 20% but it has declined to 10%. The share of undifferentiated (large cell) LC has also been around 10%. Clinical stage at LC diagnosis in Sweden has been earlier IV (metastatic) in >60% of the patients but its share has declined to 50%, when stage I had climbed to over 20% past stage III (below 20%).^8^ In small cell LC stage IV has remained at over 70% (20230921_nlcr_nationell_rapport2022.pdf [cancercentrum.se]).

Familial risk of LC (i.e., two or more first‐degree relatives diagnosed with LC because of shared smoking habits, genes or other shared reasons) is well known and the relative risk estimates are about 2.0 between parents and offspring.^9^, ^10^, ^11^, ^12^ Sex‐specific data are rare and should gauge the changes in prevalence of male and female smokers. Similarly, histology‐specific family studies are rare and as histology has changed during the past decades it is relevant to repeatedly assess its possible influence on familial risk.^13^, ^14^, ^15^ LC is a multifactorial disease in which genes and environment interact in a complex way, which is of relevance to familial risk.^16^ In the present study, we cover familial risks in LC with focus on sex‐ and time‐related changes correlating with histological changes using the unique family resources of Sweden.^17^

## 2 METHODS

The most recent update of the Swedish Cancer Registry (1961–2021) was used in the study. The Registry is nation‐wide with high coverge of cases except for some very old age cancers.^18^, ^19^ Family relationships were obtained from the Multigeneration Register, containing the current and past Swedish population in families and including 16 million individuals.^20^ ‘The offspring generation’ was born after 1931 and by 2021, the oldest offspring reached the age of 90 years; the siblings could be defined only in the offspring generation. The parental generation was the biological parents of the offspring, the oldest of whom were born in the 1800s.^20^ The linkages between family members and their cancer diagnoses were done through the unique personal identification number. The family study was restricted to offspring born in Sweden and with complete linkages to both parents.

LC cases were identified based on ICD‐7 classification, which was available across the study period. The codes for LC were 162 and 163 but as our study was focused on risk in primary LC, only cases labelled as 1621‐‘lung cancer specified as primary’ were considered. The families where any member was first diagnosed with LC not specified as primary were excluded from analysis.

Histology was available through the whole study period. We focused on four main types identified based on 3‐digit pathological anatomical diagnosis (PAD) code: adenocarcinoma 096, squamous cell carcinoma 146 and code 196 labelled as ‘unspecified’ until 1985, after which it was divided into small cell carcinoma 186 and large cell carcinoma 196. These codes were used before the ICD‐O system was introduced. The analysis of risks for small cell and large cell carcinoma was restricted to period after 1990. The probands with multiple histologically distinct LCs were excluded from histology‐specific analysis.

Familial risks of LC were calculated for an offspring/sibling case when his parent or co‐sibling was a proband diagnosed with LC. In age‐specific analysis, only the case age was defined. Familial risks were estimated based on the standardized incidence ratio (SIR) which is the ratio of observed number of cases in population at risk to expected number of cases. The observed number corresponds to number of cancers in population whose first degree relative was diagnosed with LC. The expected number of cases was calculated based on cancer incidence in offspring population without first degree relative (parent or sibling) with LC. The follow‐up started on date of birth or beginning of study (1st January 1961), whichever came later. The follow‐up was terminated at time of death, emigration, LC diagnosis or end of study (29th December 2021), whichever came earliest. The rates were standardized based on sex, age (5‐year groups), calendar period (5‐year groups), educational level (<9 years, 9 years, 10–11 years, 12 years, college <3 years, university graduate, postgraduate) and geographic region (north, south and 3 largest cities). The 95% confidence intervals (CIs) were calculated assuming that observed rates follow Poisson distribution. In the sibling risk analysis, variance was corrected for dependence of observations.^21^ We considered SIR estimates to be different when their 95% CIs were non‐overlapping.

The incidence rates by calendar year (Figures S1 and S2) were calculated as number of lung cancer cases divided by person‐time at risk. The age‐standardized rates were estimated as weighted mean (based on the World Standard Population) of age‐specific incidence rates (5‐year age groups).

All statistical analyses and data visualization were done using SAS and R (version 4.4.0).

## 3 RESULTS

### 3.1 Incidence by histological types

Age‐standardized (world) male and female incidence for all LC for period 1961 to 2021 are described in Figure S1. Male incidence culminated in 1982 followed by a decline by 50% to 2021. Female incidence culminated in 2018, having crossed male incidence a few years earlier. We show additionally concurrent literature estimates of smoking prevalence among Swedish men (since 1951) and women (since 1963) reproduced from Lee et al.^22^ Note that no lag time was considered between smoking prevalence and LC risk.

Age‐standardized (world) male histology‐specific incidence is shown in Figure S2A. Among the histological types unspecified carcinoma reached the peak incidence (11/100,000) in the early 1970s. The histological “entity” was divided in 1986, in response to the WHO 1981 reclassification, to include large cell carcinoma and small cell carcinoma. Male SCC incidence declined from its peak at around 1980 while female SCC was always rare. Adenocarcinoma increased continuously up to its peak before 2020. For women (Figure S2B) adenocarcinoma increased up to 2015, large cell carcinoma decreased steeply after 2010 and the other histologies remained constant. The situation in 2021 was that adenocarcinoma accounted for a half of all main subtypes for male and more than 60% for female LC cases.

### 3.2 Familial case numbers and proportions

During the study period from 1961 to 2021 a total of 26,075 male and 28,510 female LCs were recorded. Among male LC patients 14.6% had a first‐degree relative diagnosed with LC; for females the familial proportion was 16.2%.

### 3.3 Familial risks

Familial risk was always calculated for the case (offspring/sibling) whose proband (parent/sibling) was diagnosed with LC.

**FIGURE 1:**
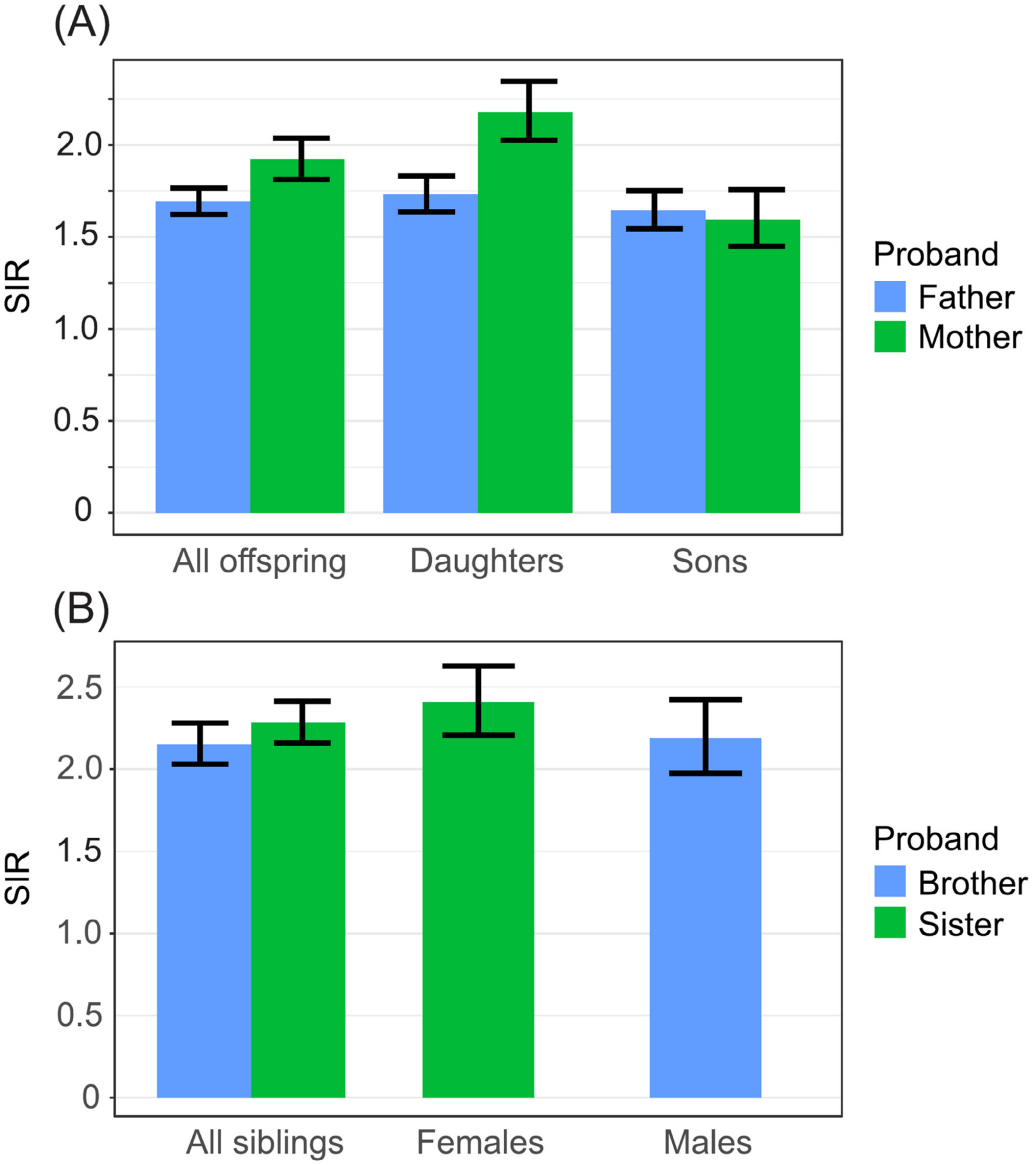
Sex‐specific familial risks with 95% CIs for lung cancer in offspring of fathers and mothers (A) and in siblings (B).

Sex‐specific familial risks are shown in Figure 1. The risk for maternal offspring was higher than for paternal offspring (maternal: SIR = 1.9 [1.8–2.0], *N* = 1133; paternal: SIR = 1.7 [1.6–1.8], *N* = 2162), particularly for mother‐daugthter (SIR = 2.2 [2.0–2.3], *N* = 716) than father‐son (SIR = 1.6 [1.5–1.8], *N* = 962) pairs (Figure 1A). Risk for sons was equally transmitted from either parent (mother‐son: SIR = 1.6 [1.5–1.8], *N* = 417). Among siblings, sister pairs had a somewhat higher risk (SIR = 2.4 [2.2–2.6], *N* = 977) compared to brother pairs (SIR = 2.2 [2.0–2.44], *N* = 717) (Figure 1B).

**FIGURE 2:**
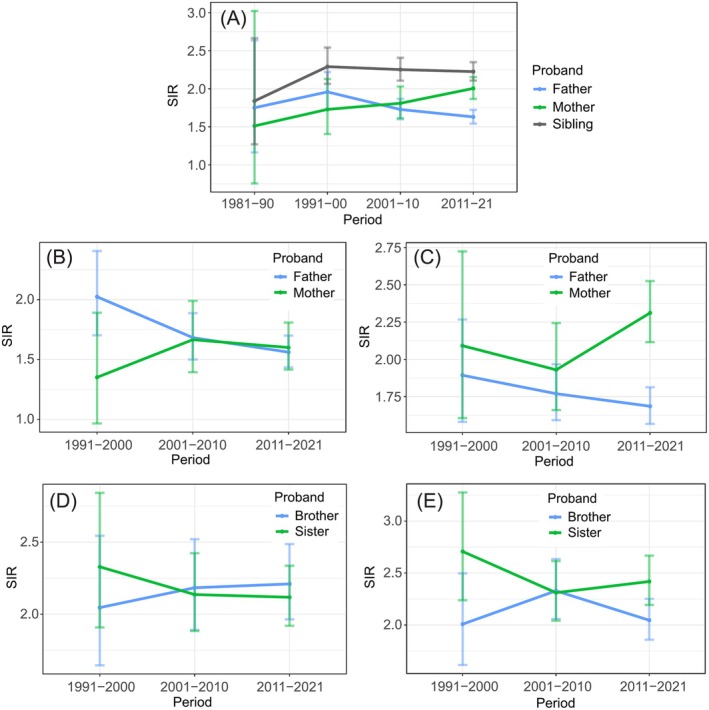
Temporal trends for lung cancer (SIRs with 95% CIs) when father, mother or sibling was diagnosed with lung cancer (A); when sons (B) or daughters (C) were diagnosed with their parents; when males (D) and females (E) were diagnosed with their siblings.

In Figure 2, we show periodic SIR trends when only father, mother or sibling was a proband diagnosed with LC but the sex of the case was not defined (panel A). Numbers of familial cases were few in the early period and thus wide 95%CIs. Risks between siblings were highest at all time points and from 1991 to 2000 onward SIRs varied between 2.2 and 2.3. Risks increased steadily for offspring, from 1.5 [0.8–3.0] (*N* = 8) to 2.0 [1.9–2.2] (*N* = 745), when mothers were probands. For offspring of father probands the risk decreased from 2.0 [1.7–2.2] (*N* = 246) to 1.6 [1.5–1.7] (*N* = 1255). Thus, overall familial risks remained constant since the 1990s. In panel B, the case sex was male (son); his SIR decreased when the father was a proband and oppositely increased when the mother was a proband; for both the final SIR was similar (father‐son: SIR = 1.6 [1.4–1.7], *N* = 531; mother‐son: SIR = 1.6 [1.4–1.8], *N* = 256). In panel C, the case sex was female (daughter); her SIR steadily declined to 1.7 [1.6–1.8] (*N* = 724) when father was a proband and increased to 2.3 [2.1–2.5] (*N* = 489) when mother was a proband. The risk between siblings was assessed in panel D (male case) and E (female case). In panel D, the SIR stay at about 2.2 and in E the final SIR for sister pairs was 2.4 [2.2–2.7] (*N* = 554) compared to 2.0 [1.9–2.3] (*N* = 417) for sister–brother pairs.

**FIGURE 3:**
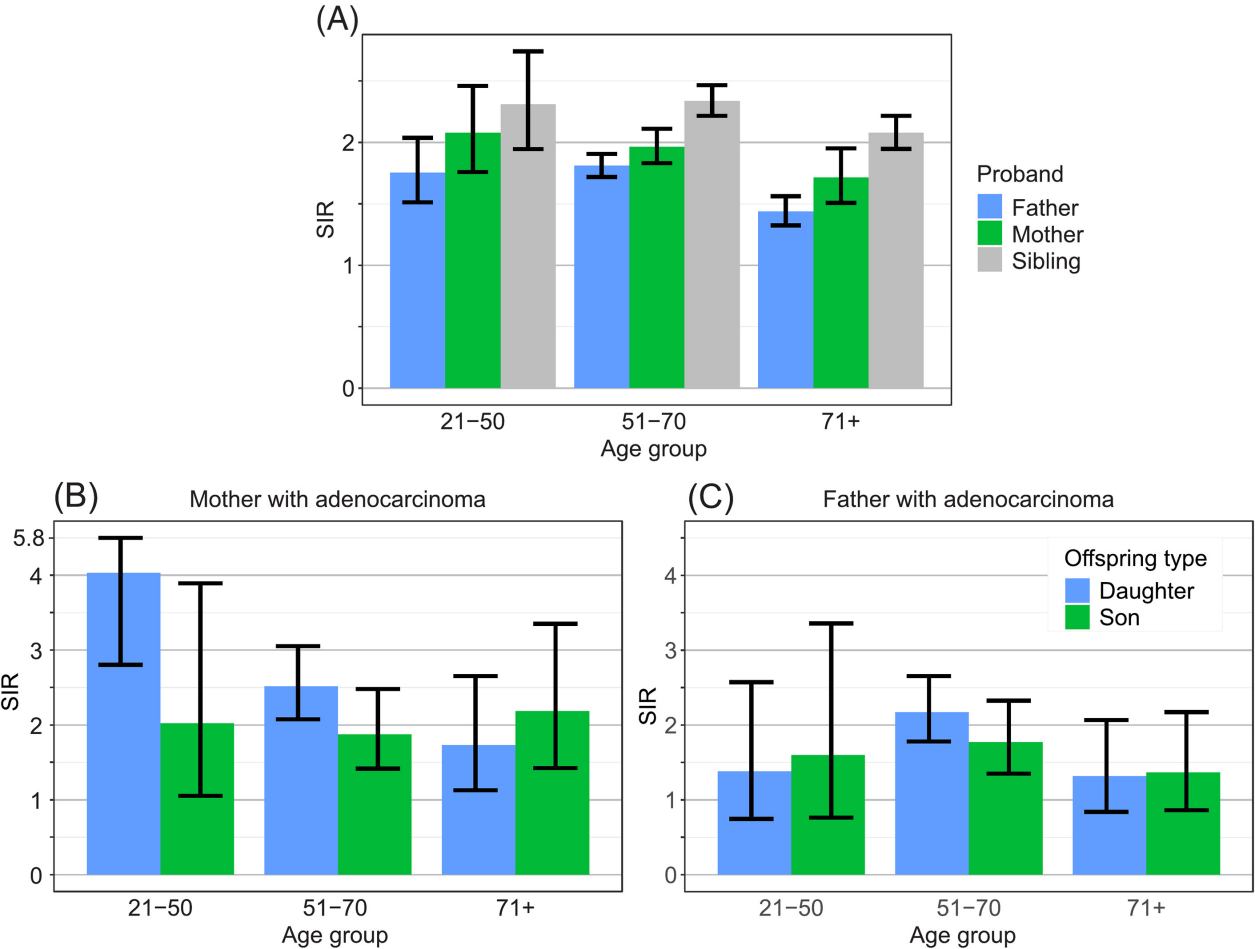
Familial risks in all lung cancer (A) and in only adenocarcinoma (B, C) by age and sex; in (B) mother and in (C) father as well as their offspring were diagnosed with adenocarcinoma.

Figure 3 shows age‐group specific familial SIRs for offspring whose parents/siblings were diagnosed with LC. In panel A, total LC was considered, and in all age groups SIRs increased in proband order sibling>mother>father. However, in each of the proband groups SIRs were almost identical in the youngest (21–50 year) and the next (51–70 year) age group. The decline in SIR was first among those older than 70 years. In panels B and C, age‐ and sex‐specific SIRs are shown for adenocarcinoma only. In general, maternal offspring had higher risks than paternal ones but the only difference was at age 21–50 years for maternal daughter (SIR = 4.0 [2.8–5.8], *N* = 29) compared to paternal daughters (SIR = 1.4 [0.7–2.6], *N* = 10). We do not show similar analysis for SCC or other histological types because of low case numbers.

**FIGURE 4:**
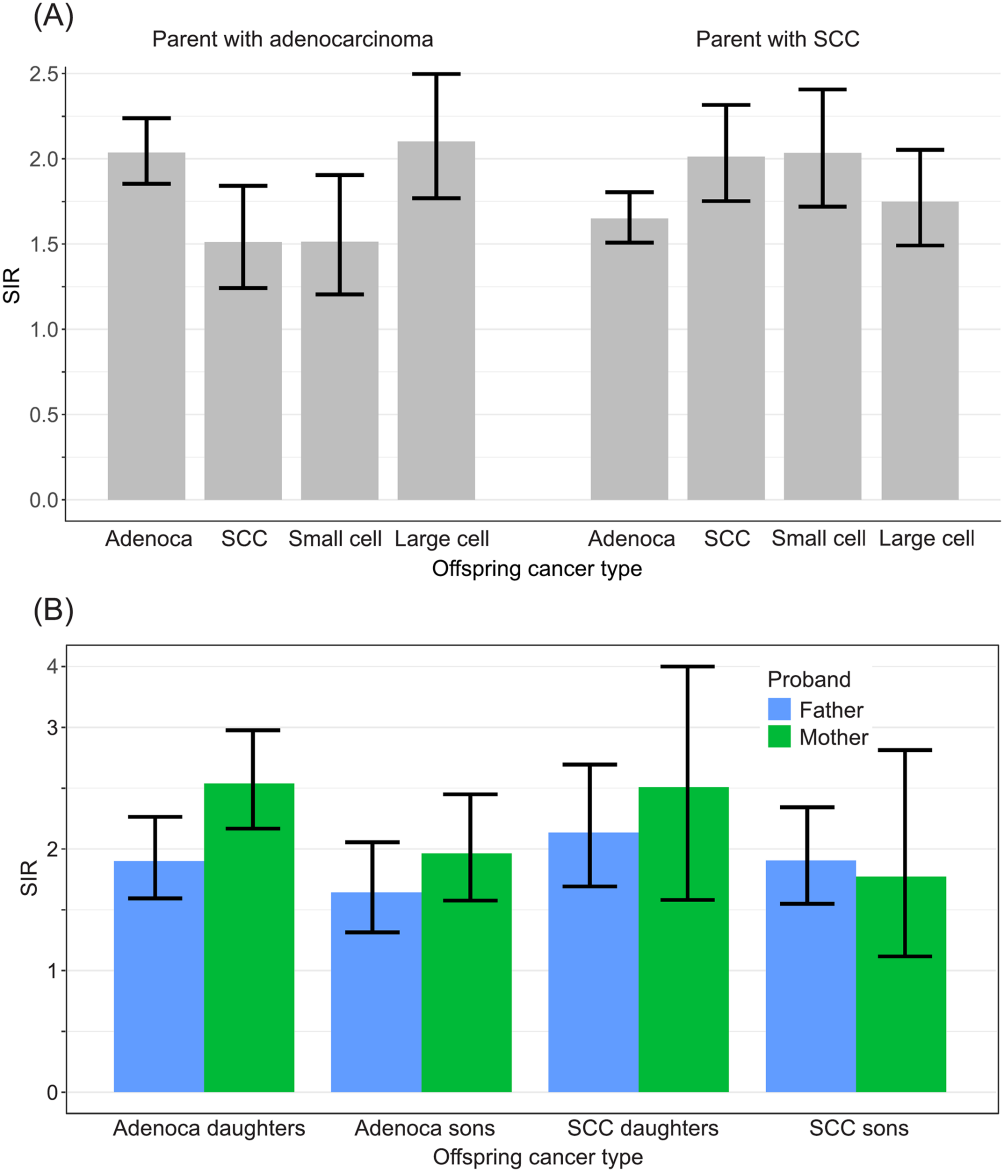
(A) Histology‐specific risks for lung cancer in offspring when single parent (and no sibling) was diagnosed with adenocarcinoma or SCC. (B) Histology‐ and sex‐specific risks for concordant cancer (the same histological type) in offspring when single parent (and no sibling) is diagnosed with adenocarcinoma or SCC.

Histology‐specific risks in offspring when only one parent was diagnosed with LC (adenocarcinoma or SCC) and no other sibling was affected are shown in Figure 4. In panel A, by parental adenocarcinoma, offspring adenocarcinoma risk was 2.0 [1.9–2.2] (*N* = 434) and large cell carcinoma risk was 2.1 [1.8–2.5] (*N* = 129). The adenocarcinoma‐adenocarcinoma association was higher than the adenocarcinoma‐SCC association (SIR = 1.5 [1.2–1.8], *N* = 99). In the offspring of parents with SCC, the relative risks were highest for SCC (SIR = 2.0 [1.8–2.3], *N* = 197) and small cell carcinoma (SIR = 2.0 [1.7–2.4], *N* = 136). In panel B, sex was additionally considered for concordant adenocarcinoma and SCC. Curiously, all associations with daughters were higher than those with sons and the relative risk for daughters of mothers with adenocarcinoma (SIR = 2.5 [2.2–3.0], *N* = 153) was higher than those for sons of fathers with adenocarcinoma (SIR = 1.6 [1.3–2.1], *N* = 77). Similarly, higher risks were observed for females in SCC (mother‐daughter SIR = 2.5 [1.6–4.0], *N* = 18; father‐son SIR = 1.9 [1.5–2.3], *N* = 90), yet without clear differences.

**FIGURE 5:**
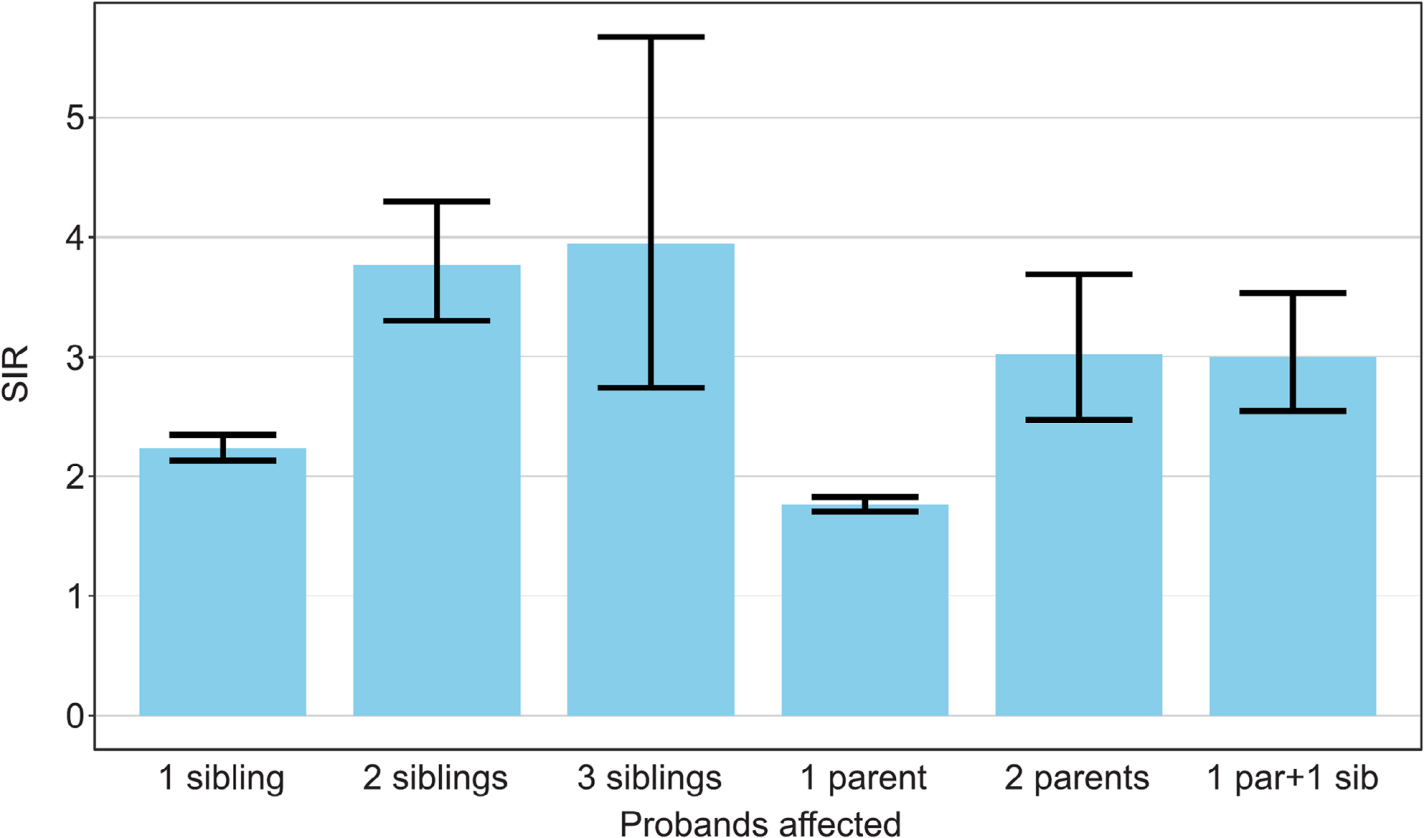
Risk of lung cancer in sibling/offspring when increasing numbers of sibling/parents were probands.

Results in families with multiple affected patients are shown in Figure 5. The SIRs for siblings increased from 2.2 [2.1–2.3] (*N* = 3215) when one other sibling was diagnosed with LC to 3.8 [3.3–4.3] (*N* = 332) when two other siblings were diagnosed and to 3.9 [2.7–5.7] (*N* = 39) when three other siblings were diagnosed. SIRs for offspring increased from 1.8 [1.7–1.8] (*N* = 3295) when only one parent was affected to 3.0 [2.5–3.7] (*N* = 96) when two parents were affected. The risk was equally high (SIR = 3.0 [2.5–3.5], *N* = 280) when a parent and a sibling were affected.

**FIGURE 6:**
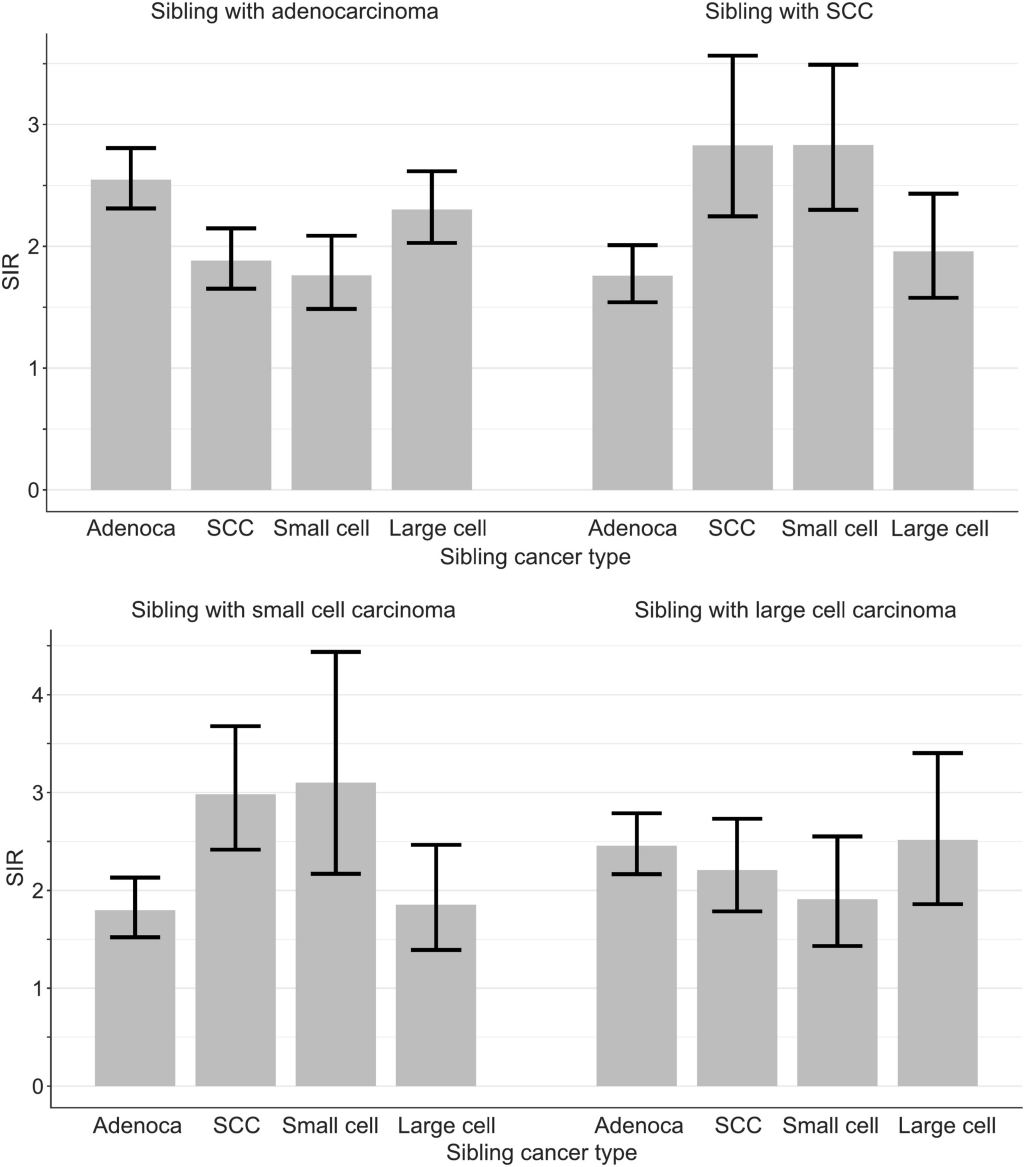
Histology‐specific risks for lung cancer in siblings, when one sibling (proband) was diagnosed with adenocarcinoma, SCC, small cell carcinoma or large cell carcinoma (no parent with lung cancer) and the co‐sibling was diagnosed histology specific lung cancer.

In Figure 6, histology‐specific risks are shown for the second sibling when one sibling was diagnosed with histology‐specific LC. The overall observation was that for each four histological types the concordant association between the siblings was always highest but even all discordant associations were significant (lower CIs well above SIR 1.0). For siblings of probands with adenocarcinoma, the highest relative risks were for adenocarcinoma (SIR = 2.5 [2.3–2.8], *N* = 789) and large cell carcinoma (SIR = 2.3 [2.0–2.6], *N* = 236); the SIR for concordant adenocarcinoma was significantly higher than those for SCC (SIR = 1.9 [1.7–2.1], *N* = 223) or small cell carcinoma (SIR = 1.8 [1.5–2.1], *N* = 133). Conversely, when one sibling was diagnosed with SCC, the second sibling had highest relative risk for concordant SCC (SIR = 2.8 [2.2–3.6], *N* = 138) and small cell‐carcinoma (SIR = 2.8 [2.3–3.5], *N* = 88), which were significantly higher than the risk for adenocarcinoma (SIR = 1.8 [1.5–2.0], *N* = 218). When one sibling was diagnosed with small cell carcinoma the SIR for second sibling for concordant cancer was 3.1 [2.2–4.4] (*N* = 59), higher than for second sibling with adenocarcinoma (SIR = 1.8 [1.5–2.1], *N* = 135). When the proband was diagnosed with large cell carcinoma the risk for concordant disease was 2.5 [1.9–3.4] (*N* = 82) and the SIRs varied between 1.9 and 2.5 for all discordant associations.

## 4 DISCUSSION

Cigarette smoking is not the only cause of LC but its contribution is overwhelming.^3^ Thus though Swedish men have had the lowest smoking prevalence in Europe, only 12% of diagnosed LCs in 2022 were among never smokers (women 15%) (https://cancercentrum.se/samverkan/cancerdiagnoser/lunga-och-lungsack/kvalitetsregister). As novel results of the present study, we first showed that familial risks in Sweden have remained constant since the 1990s (Figure 2A) since the familial case numbers allowed a reasonably accurate assessment. However, the constant trend hid a sex‐specific trend shift which was a second novel finding (Figure 2B,C). Male familial risks (father‐son) declined from 2.0 to 1.6 while female familial risks (mother‐daughter) increased from 2.1 to 2.3. In the total follow‐up period until 2021, the female (mother‐daughter) familial risk was 2.2 compared to male (father‐son) risk of 1.6, a highly significant difference (Figure 1A). The female preference in familial risk (in Figures 1, 2, 3) points to the importance of the mother as a role model in the family, especially to the daughter. Familial risk was somewhat higher in cases diagnosed before age 70 years irrespective of the proband (father, mother, sibling) but the age gradient was not as clear as in many other familial cancers which show a clear early age component.^10^ Another novel observation with large case numbers was that the histology‐specific familial risks (parent‐offspring and between siblings) were usually highest for concordant histology but also significant for discordant histology.^11^, ^15^ However, there was a preferential clustering of familial risk between adenocarcinoma and large cell carcinoma and another clustering between SCC and small cell carcinoma.

It is possible that the sex trend in familial risk was related to the fact that male LC incidence peaked already in 1982 while that for women peaked as recently as in 2018.^6^ In particular, Figure 2 provides circumstantial evidence for changing familial risk according to smoking prevalence. Most remarkably, female familial risk doubled (from 1.5 to 2.0) between 1981–1990 and 2011–2021 when the female incidence also doubled (Figure S1). For men the decrease in familial risk was less than the actual incidence decline. In our previous study, we investigated familial association of LC with any discordant cancer.^14^ LC was associated strongly in families with seven other cancers; six of these discordant sites were known smoking related cancers. This kind of data supports the view that in Sweden familial LC is mainly smoking‐related. However, international studies show evidence that familial LC may be diagnosed also in non‐smokers. There is a series of case–control studies on non‐smokers (and ex‐smokers) from USA published in the 1990s which provided suggestive evidence on familial risks in non‐smokers and there are more recent Asian studies, as recently reviewed.^23^, ^24^, ^25^ However, the strongest evidence for familial LC among non‐smokers has been provided by the Utah Population Database where smoking information was obtained from death certificates; familial risk for nonsquamous, non‐small cell lung cancer was clearly increased among non‐smokers while SCC was not.^15^ A note should be added that according to the paper <10% of the Utah population have been smokers.^15^ That study reviews also the previous literature tackling the question of familial LC unrelated to smoking.

Familial risk depends strongly on the number of affected probands which has been reported by us and others earlier.^9^, ^10^, ^11^, ^12^ In general familial risks were higher between siblings than between parents and offspring but siblings were usually younger at diagnosis compared to parents. What has driven the changes in LC family history has remained unclear.^22^, ^26^, ^27^ Evaluation of histological classification changes over time has pointed out that as a consequence of the WHO 1981 reclassification of LC, a part of large cell carcinomas were reclassified as adenocarcinoma, and small cell carcinoma was separated as an independent entity.^28^ That study further refers to reclassification of the same LC specimens by pathologists or pathology panels with large shifts between histologies but also a general increase in adenocarcinoma.^28^ Women have had higher proportion of adenocarcinoma compared to men which has also remained unexplained and the speculation about sex differences between smoking behaviors have not been validated.^22^, ^26^, ^29^, ^30^ We speculated that there may be some genetic underpinnings to the presentation of LC histology but the present results do not support such mechanisms. However, we could demonstate that increased familial risk was shared by all histological types, although with some preferential aggregation of adenocarcinoma with large cell carcinoma and SCC with small cell carcinoma. In agreement, a small study on micro‐dissected LC samples reported that different histological types shared 70%–80% of point mutations and copy number aberrations but showed some distinct transcriptomic pathways.^31^

The dominant role tobacco smoking in LC causation has been established in several ways. Firstly epidemiological, for example using population attributable fractions of LC; an estimate from UK for year 2015 arrived at 74.1% for male and 70.2% for female active smoking.^32^ For the other considered risk factors, occupation accounted for 20.5% for men and 4.3% for women, while radiation and air pollution were estimated at around 5% for both men and women.^32^ Secondly clinically, only more than 10% of newly diagnosed LCs in Sweden are found in non‐smokers, as discussed above. Thirdly by molecular fingerprints, through genetic mutation signatures of tobacco smoke in LC patients.^33^, ^34^ Yet such mutation signatures were able to distinguish a signature in DNA of LC patients which differed from the typical tobacco‐related signature and was suggested to indicate mutations in non‐smoker LC.^25^ A recent genomic profiling study of non‐smoking LC patients showed mutation signatures suggestive of passive exposure to cigarette smoke and distinct immune transcriptional subtypes.^35^ The role of tobacco smoking in LC will diminish in due time in populations with decreasing levels of smokers, and the role of other environmental carcinogens will become more prominent, as they already are in some subpopulations exposed to agents such as arsenic, asbestos, radon or occupational carcinogens.^36^

In conclusion, the Swedish LC experience may be internationally unique because of the early (1982) culmination of the male LC incidence and the late (2018) culmination of the female LC incidence with these incidence rates crossing around 2015.^6^ We could show here a decline in male familial risk and an increase in female familial risk, which doubled at the same time as the incidence doubled. Female familial risks exceeded male ones for adenocarcinoma and SCC. These finding supported the view that familial risk depends on the background incidence levels. We could further demonstrate a distinct familial risk for each of the four histological types of LC which was higher for concordant than discordant histologies. The shared histology‐specific familial risk suggests that LC histology is not a genetic trait.

## AUTHOR CONTRIBUTIONS

**Kari Hemminki:** Conceptualization; writing – original draft; methodology; writing – review and editing; formal analysis; project administration. **Frantisek Zitricky:** Investigation; methodology; writing – review and editing; formal analysis; software. **Kristina Sundquist:** Investigation; methodology; writing – review and editing. **Jan Sundquist:** Investigation; methodology; writing – review and editing. **Asta Försti:** Investigation; writing – original draft; writing – review and editing; data curation. **Akseli Hemminki:** Investigation; writing – original draft; writing – review and editing; resources.

## FUNDING INFORMATION

The Cooperatio Program, research area SURG and National Institute for Cancer Research—NICR (Programme EXCELES, ID Project No. LX22NPO5102), funded by the European Union—Next Generation EU and the SALVAGE Project, reg. no: CZ.02.01.01/00/22_008/0004644, The Swedish Research Council, Jane and Aatos Erkko Foundation, Sigrid Juselius Foundation, Finnish Cancer Organizations and Helsinki University Central Hospital.

## CONFLICT OF INTEREST STATEMENT

Akseli Hemminki is shareholder in Circio Holdings ASA. Akseli Hemminki is employee and shareholder in TILT Biotherapeutics Ltd. The other authors declared no conflict of interest.

## ETHICS STATEMENT

The study was approved by the Regional Ethical Review Board in Lund University, February 6, 2013 (Reference 2012/795 and subsequent amendments). The Regional Ethical Review Board in Lund University waved the need to include informed consent. The study was conducted in accordance to Declaration of Helsinki.

## Supporting information


**FIGURE S1.** Incidence (ASR‐World per 100 00) of all lung cancer in men and women in Sweden from 1961 to 2021. Estimated frequeny of smokers in population aged over 20 years is shown, as cited from Reference 22.
**FIGURE S2.** Incidence (ASR‐World per 100 00) of histological subtypes of lung cancer in Sweden from 1961 to 2021. Note that code 196 (undifferentiated) was split after 1985 to small cell carcinoma (186) and large cell carcinoma (196).

## Data Availability

The data that support the findings of this study are available from the corresponding author and with permission of the Swedish health authority upon reasonable request.
