# Supplementary material for: Sex specific familial risk in lung cancer through changing histologies in Sweden
Source: Int J Cancer. 2025 Mar 29;157(5):858–66. doi: 10.1002/ijc.35431 (PMC12232509; doi:10.1002/ijc.35431)
Supplement: Supplementary file 1 — FIGURE S1. Incidence (ASR‐World per 100 00) of all lung cancer in men and women in Sweden from 1961 to 2021. Estimated frequeny of smokers in population aged over 20 years is shown, as cited from Reference 22. FIGURE S2. Incidence (ASR‐World per 100 00) of histological subtypes of lung cancer in Sweden from 1961 to 2021. Note that code 196 (undifferentiated) was split after 1985 to small cell carcinoma (186) and large cell carcinoma (196). [file IJC-157-858-s001.pdf]

SUPPLEMENTARY MATERIAL

SEX SPECIFIC FAMILIAL RISK IN LUNG CANCER THROUGH CHANGING HISTOLOGIES IN SWEDEN

Kari Hemminki , Frantisek Zitricky, Kristina Sundquist, Jan Sundquist, Asta Försti, Akseli Hemminki

Table of contents:

Supplementary Figure 1. Incidence (ASR-World per 100 00) of all lung cancer in men and women in Sweden from 1961 to 2021. Estimated frequeny of smokers in population aged over 20 years is shown, as cited from ref. (22).

Supplementary Figure 2. Incidence (ASR-World per 100 00) of histological subtypes of lung cancer in Sweden from 1961 to 2021. Note that code 196 (undifferentiated) was split after 1985 to small cell carcinoma (186) and large cell carcinoma (196).

Suppl. Fig. 1

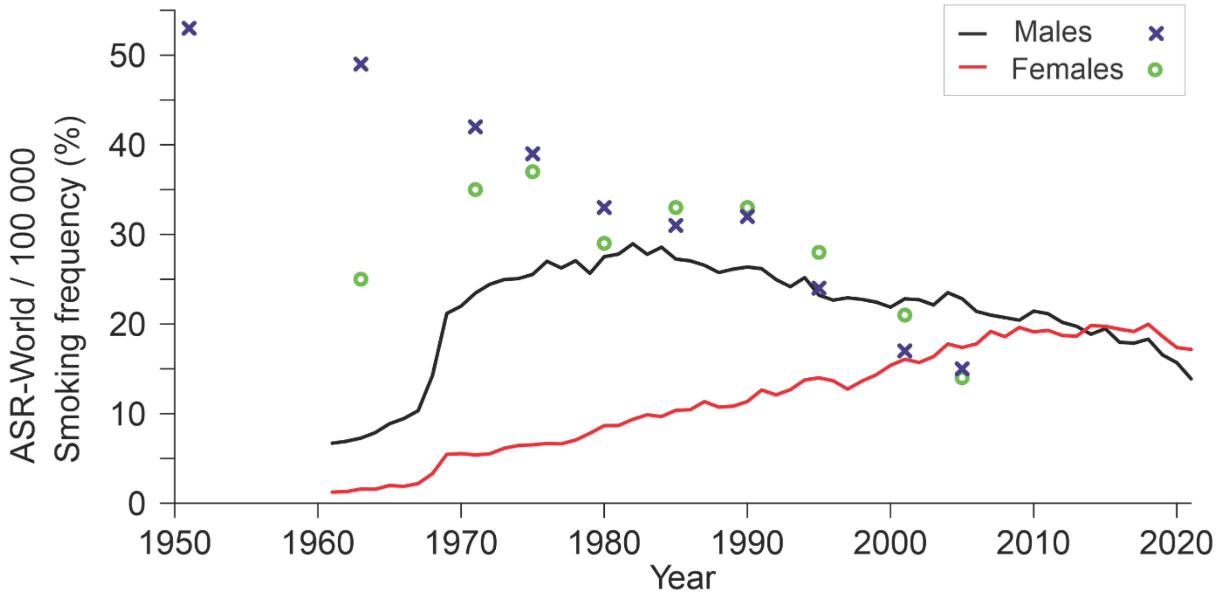

Solid lines: age-standardized (World) male and female incidence of all lung cancers for period 1961-2021. Individual points show the estimated frequency of smokers in the population older than 20 years in Sweden (data collected from Lee et al. 2012).

Suppl. Fig. 2

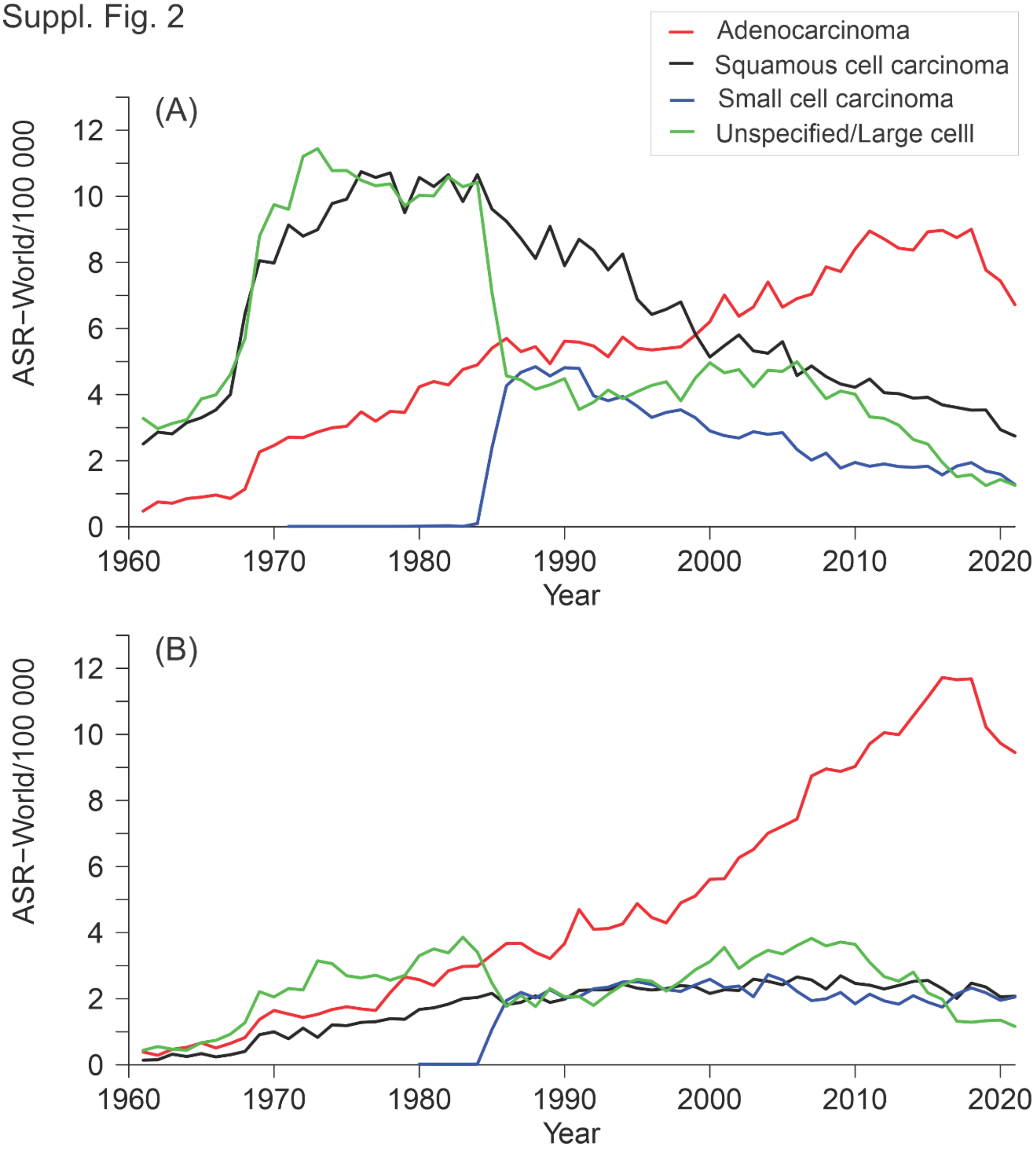

Age standardized (World) male (A) and female (B) histology-specific incidence of lung cancer in Sweden.
